# Supplementary material for: Cas9 exo-endonuclease eliminates chromosomal translocations during genome editing
Source: Nat Commun. 2022 Mar 8;13:1204. doi: 10.1038/s41467-022-28900-w (PMC8904484; doi:10.1038/s41467-022-28900-w)
Supplement: Supplementary file 1 — Supplementary Information [file 41467_2022_28900_MOESM1_ESM.pdf]

SUPPLEMENTARY INFORMATION

Cas9 exo-endonuclease eliminates chromosomal translocations during genome editing

Yin et al

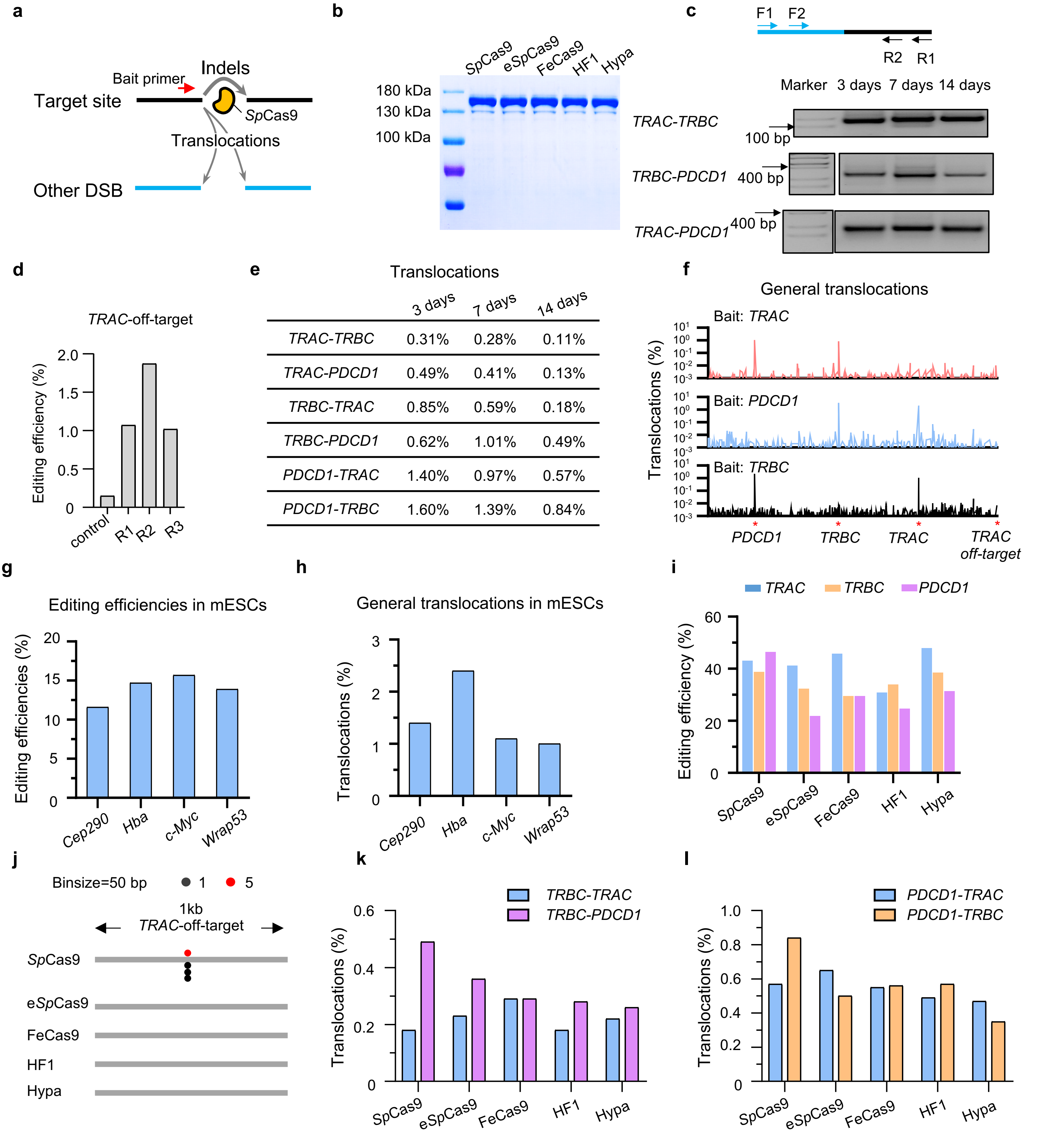

Supplementary Figure 1. Translocations form during genome editing in human T cells and mESCs

a. Schematics for the editing outcomes of CRISPR-Cas9 identified by PEM-seq. **b**. Purification of *Sp*Cas9 and *Sp*Cas9 high-fidelity variants by SDS-PAGE. **c**. Detection of translocations among *TRAC*, *TRBC*, and *PDCD1* by nested PCR in human T cells at 3-, 7- and 14-days post-transfection. F1 and R1 were used in the first round of PCR, and products were recycled for the second round of PCR with F2 and R2. DNA markers are shown on the left. Details are in the **Methods**. **d**. Editing efficiency for *TRAC* off-targets in human T cells 3-days post-transfection detected by PEM-seq (N=3). **e**. Average percentages for the indicated translocations detected by PEM-seq in human T cells at 3-, 7- and 14-days post-transfection. **f**. Genome-wide patterns of translocations cloned from *TRAC*, *TRBC,* or *PDCD1* by PEM-seq in human T cells at 3-days post-transfection. Red asterisks indicate the *TRAC*, *TRBC*, *PDCD1* on-targets and the identified *TRAC* off-target. **g** and **h**. Editing efficiency (**g**) and percentage of general translocations (**h**) cloned from *Cep290*, *Hba*, *c-Myc*, and *Wrap53* by PEM-seq in mESCs 3-days post-transfection. **i**. Editing efficiency for *Sp*Cas9 and the indicated high-fidelity variants at *TRAC*, *TRBC*, and *PDCD1* loci detected by PEM-seq at 3-days post-transfection. The *Sp*Cas9 library was one of the three replicates shown in Figure 1b. **j**. The distributions of translocation junctions at the identified *TRAC* off-target for *Sp*Cas9 and the indicated high-fidelity variants in human T cells at 3-days post-transfection. **k** and **l**. Percentages of *TRBC-TRAC, TRBC-PDCD1* (**k**), *PDCD1-TRAC* and *PDCD1-TRBC* (**l**) translocations induced by *Sp*Cas9 and the indicated variants detected by PEM-seq in human T cells cloned from *TRAC* 3 days post-transfection, N=1. Source data are provided as a Source Data file.

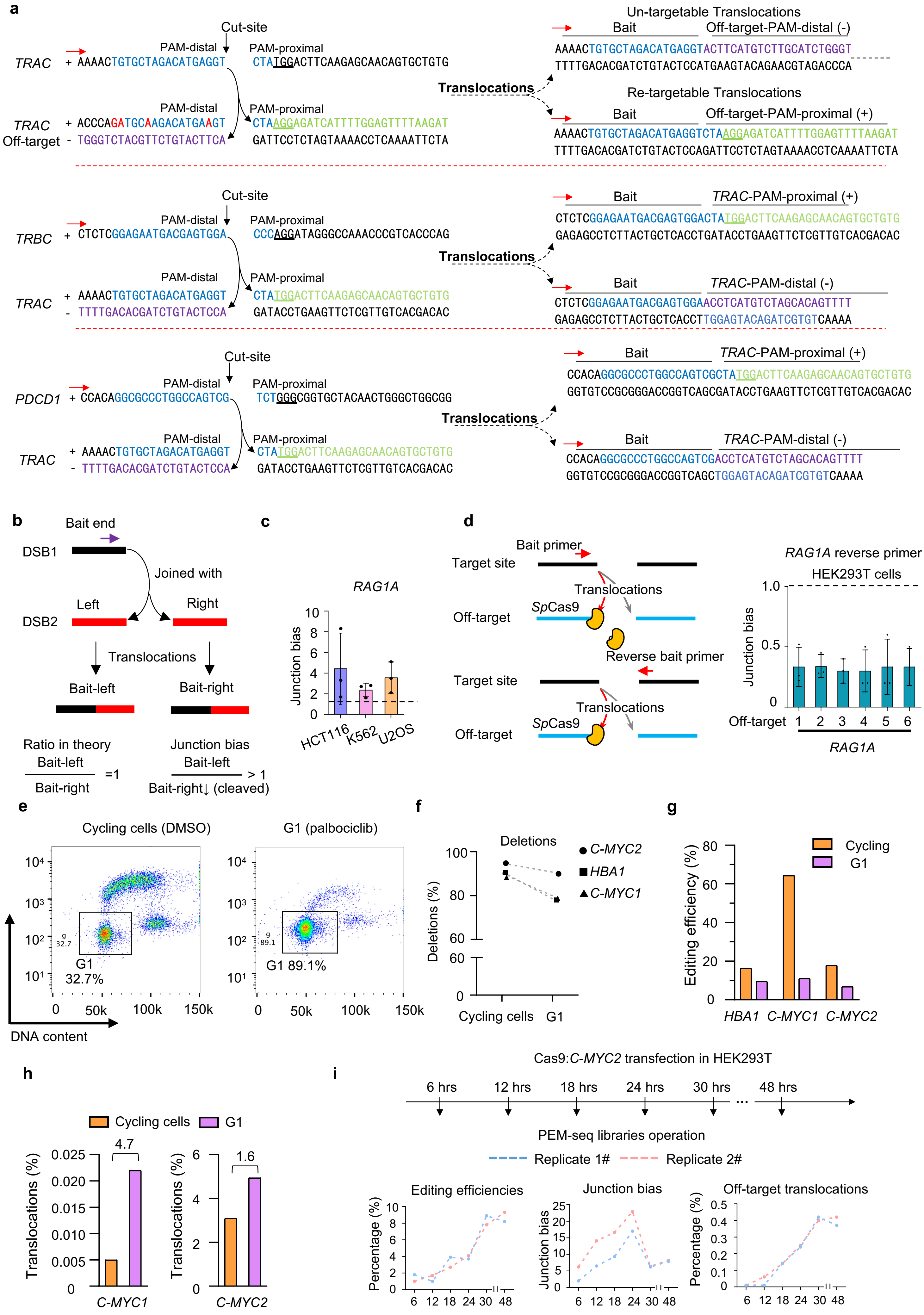
a
Cut-site
PAM-distal
PAM-proximal
TRAC
+ AAAACTGTGCTAGACATGAGGT
CTATGGACTTCAAGAGCAACAGTGCTGTG
TRAC Off-target
+ ACCCAGATGCAAGACATGAAGT
- TGGGTCTACGTTCTGTACTTCA
CTAAGGAGATCATTTTGGAGTTTTAAGAT
GATTCCTCTAGTAAAACCTCAAAATTCTA
Translocations
Un-targetable Translocations
Bait
Off-target-PAM-distal (-)
AAAACTGTGCTAGACATGAGGTACTTCATGTCTTGCATCTGGGT
TTTTGACACGATCTGTACTCCATGAAGTACAGAACGTAGACCCA
Re-targetable Translocations
Off-target-PAM-proximal (+)
AAAACTGTGCTAGACATGAGGTCTAAGGAGATCATTTTGGAGTTTTAAGAT
TTTTGACACGATCTGTACTCCAGATTCCTCTAGTAAAACCTCAAAATTCTA
TRBC
+ CTCTCGGAGAATGACGAGTGGA
CCCAGGATAGGGCCAAACCCGTCACCCAG
TRAC
+ AAAACTGTGCTAGACATGAGGT
- TTTTGACACGATCTGTACTCCA
CTATGGACTTCAAGAGCAACAGTGCTGTG
GATACCTGAAGTTCTCGTTGTCACGACAC
TRAC-PAM-proximal (+)
CTCTCGGAGAATGACGAGTGGACTATGGACTTCAAGAGCAACAGTGCTGTG
GAGAGCCTCTTACTGCTCACCTGATACCTGAAGTTCTCGTTGTCACGACAC
TRAC-PAM-distal (-)
CTCTCGGAGAATGACGAGTGGAACCTCATGTCTAGCACAGTTTT
GAGAGCCTCTTACTGCTCACCTTGGAGTACAGATCGTGTCAAAA
PDCD1
+ CCACAGGCGCCCTGGCCAGTCG
TCTGGGCGGTGCTACAACTGGGCTGGCGG
TRAC
+ AAAACTGTGCTAGACATGAGGT
- TTTTGACACGATCTGTACTCCA
CTATGGACTTCAAGAGCAACAGTGCTGTG
GATACCTGAAGTTCTCGTTGTCACGACAC
CCACAGGCGCCCTGGCCAGTCGCTATGGACTTCAAGAGCAACAGTGCTGTG
GGTGTCCGCGGGACCGGTCAGCGATACCTGAAGTTCTCGTTGTCACGACAC
CCACAGGCGCCCTGGCCAGTCGACCTCATGTCTAGCACAGTTTT
GGTGTCCGCGGGACCGGTCAGCTGGAGTACAGATCGTGTCAAAA
b
Bait end
DSB1
Joined with
Left
Right
DSB2
Translocations
Bait-left
Bait-right
Ratio in theory
Bait-left / Bait-right =1
Junction bias
Bait-left / Bait-right↓ (cleaved) > 1
c
RAG1A
Junction bias
HCT116
K562
U2OS
d
Bait primer
Target site
Translocations
Off-target
SpCas9
Reverse bait primer
RAG1A reverse primer
HEK293T cells
Junction bias
Off-target
RAG1A
e
Cycling cells (DMSO)
G1 (palbociclib)
BrdU
DNA content
G1
32.7%
G1 89.1%
f
Deletions
Deletions (%)
C-MYC2
HBA1
C-MYC1
Cycling cells
G1
g
Editing efficiency (%)
Cycling
G1
HBA1
C-MYC1
C-MYC2
h
Cycling cells
G1
Translocations (%)
4.7
1.6
C-MYC1
C-MYC2
i
Cas9:C-MYC2 transfection in HEK293T
6 hrs
12 hrs
18 hrs
24 hrs
30 hrs
48 hrs
PEM-seq libraries operation
Replicate 1#
Replicate 2#
Editing efficiencies
Percentage (%)
Junction bias
Off-target translocations

Supplementary Figure 2. Junction bias caused by repeated cleavage of CRISPR-Cas9

a. DNA sequences of *TRAC*, *TRAC* off-target and PEM-seq-identified translocations between the *TRAC* on- and off-target, *TRBC* and *TRAC* and *PDCD1* and *TRAC*. Red letters indicate mismatches between the *TRAC* on- and off-target. Green nucleotides indicate the PAM-proximal end for *TRAC* off-target and purple nucleotides indicate the PAM-distal end for *TRAC* off-target. Red arrow indicates the orientation and position of bait primers for PEM-seq. **b**. Schematic illustrations of chromosomal translocations between two DSBs. In theory, the two broken ends from the same DSB2 have the equal chance to form translocations with a third broken end from DSB1. The ratio should be 1. If the bait-right translocations are cleaved multiple times, the ratio will be >1. **c**. Off-target translocation bias between on-target and off-targets of *RAG1A* in HCT116, U2OS, and K562 cells detected by PEM-seq. Mean ± SD from three replicates. The dashed line indicates the ratio=1. Note that translocation junctions from all identified off-target sites were combined to perform the analysis due to the presence of relatively few off-target translocation junctions in these cell lines. **d**. Schematics showing the generation of PEM-seq libraries with two opposite primers and junction bias detected with the reverse primer PEM-seq library. *Sp*Cas9 (yellow) can persist at the sgRNA-containing broken ends. Red arrows indicate the bait primers for PEM-seq. Mean ± SD from three replicates. **e**. Cell cycle analysis for K562 cycling cells and G1-arrested cells with BrdU and 7-AAD double staining by FACS. Black gates indicate the G1 phase. **f** and **g**. Deletion ratios (**f**) and editing efficiency (**g**) of *Sp*Cas9 targeting *HBA1*, *C-MYC1,* and *C-MYC2* sites in K562 cycling cells and G1 cells detected by PEM-seq. **h**. Percentages of off-target translocations for *Sp*Cas9 targeting *C-MYC1* and *C-MYC2* in K562 cycling cells and G1 cells detected by PEM-seq. Fold changes are at the top of the bars. **i**. Editing efficiency, junction bias at off-targets, and percentages of off-target translocations at the *C-MYC2* locus at the indicated time points in HEK293T cells detected by PEM-seq for two replicates. Source data are provided as a Source Data file.

a

Junctions for *HBA1* off-target

	*Sp*Cas9	T2A-TREX2	Cas9X2	Cas9X2d
OT1	348/43	271/82	41/34	116/28
**Bias**	**8.1**	**3.2**	**1.2**	**4.1**
OT2	251/52	312/80	7/2	83/26
**Bias**	**4.8**	**3.9**	*	**3.2**
OT3	313/75	152/65	39/39	79/42
**Bias**	**4.2**	**2.3**	**1.0**	**1.9**
Editing events	36k	154k	198k	17k

b

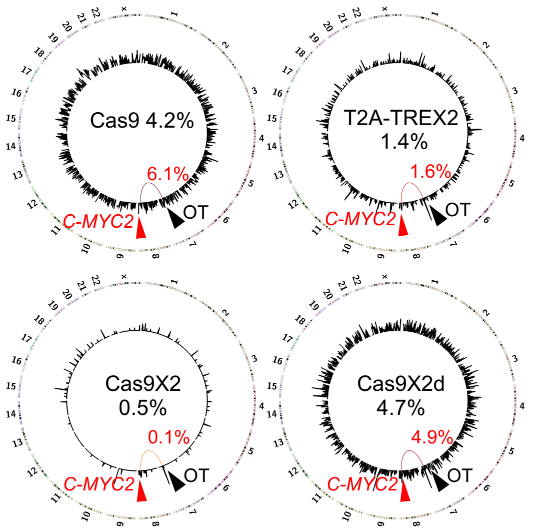

c **d**

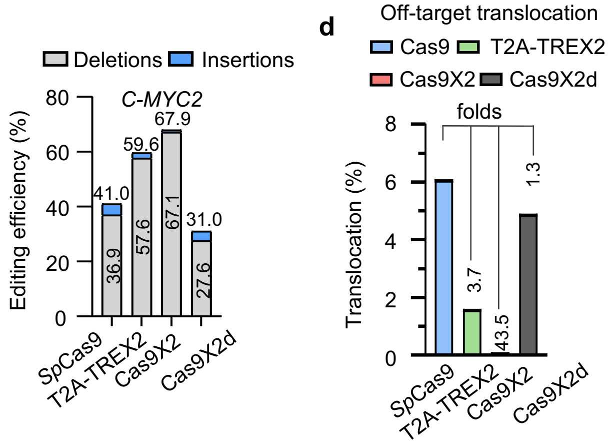

e

Junctions for *C-MYC2* off-target

	*Sp*Cas9	T2A-TREX2	Cas9X2	Cas9X2d
OT1	3,212/991	1,493/500	103/57	2,123/624
**Bias**	**3.2**	**3.0**	**1.8**	**3.4**
Editing events	78k	126k	114k	64k

f **g**

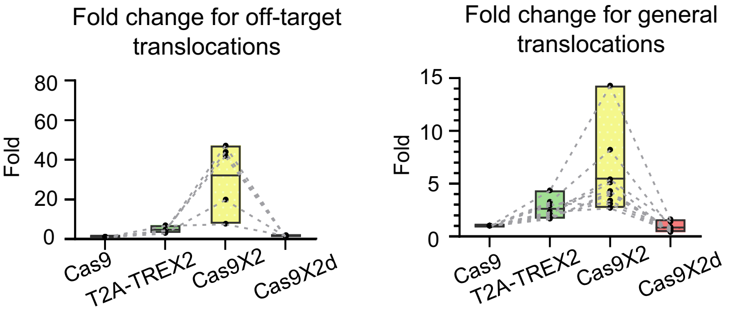

Supplementary Figure 3. Cas9X2 reduces chromosomal translocations

a. Junction bias for *Sp*Cas9, T2A-TREX2, Cas9X2, and Cas9X2d at the *HBA1* locus in HEK293T cells detected by PEM-seq. Junction numbers of identified off-targets and the calculated bias are shown, and the total numbers of editing events are shown at the bottom. Red numbers highlight sites with too few junctions. **b**. Circos plot showing the distribution of genome-wide translocations cloned from *C-MYC2* by PEM-seq in HEK293T cells, depicted as described in the legend to **Figure 3g**. **c-e**. Editing efficiency (**c**), percentages of off-target translocations (**d**), and junction bias (**e**) for *Sp*Cas9, T2A-TREX2, Cas9X2, and Cas9X2d cloned from *C-MYC2* by PEM-seq in HEK293T cells. **f** and **g**. Fold change for off-target translocations (**f**) and general translocations (**g**) of T2A-TREX2, Cas9X2, and Cas9X2d compared to *Sp*Cas9 in HEK293T cells detected by PEM-seq, one independent PEM-seq library for each locus, N=10. The loci used were *DNMT1-1*, *DNMT1-2*, *EMX1*, *HBA1*, *C-MYC1*, *C-MYC2*, *C-MYC3*, *RAG1A*, *RAG1B,* and *RAG1C*. Note that off-target junctions were not detected in several loci for Cas9X2. Values from minimum to maximum are shown in the box. The line represents the average. Source data are provided as a Source Data file.

a

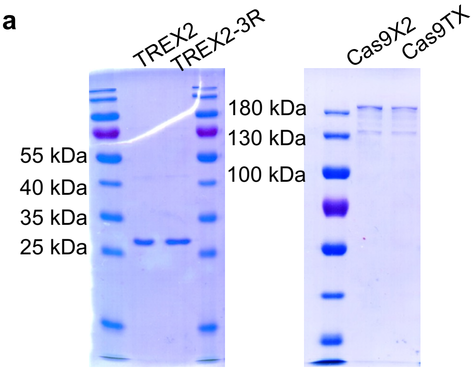

b

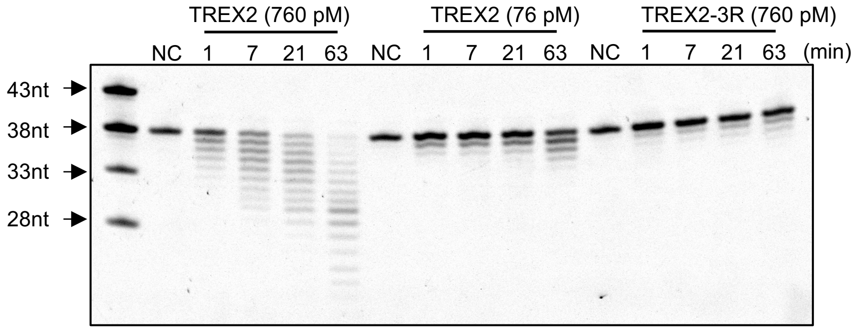

c

Junctions for *HBA1* off-target-1

	*Sp*Cas9	Cas9X2	Cas9TX
OT1	1,004 / 118	67 / 39	7 / 2
**Bias**	**8.5**	**1.7**	*
Editing events	100k	206k	124k

Junctions for *HBA1* off-target-2

	*Sp*Cas9	Cas9X2	Cas9TX
OT2	810 / 102	7 / 8	5 / 2
**Bias**	**7.9**	*	*
Editing events	100k	206k	124k

Junctions for *HBA1* off-target-3

	*Sp*Cas9	Cas9X2	Cas9TX
OT3	1,872 / 253	95 / 33	28 / 18
**Bias**	**7.4**	**2.9**	**1.6**
Editing events	100k	206k	124k

d

Junctions for *C-MYC2* off-target

	*Sp*Cas9	Cas9X2	Cas9TX
OT1	9,484 / 2,022	233 / 76	210 / 86
**Bias**	**4.7**	**3.1**	**2.4**
Editing events	170k	180k	220k

e

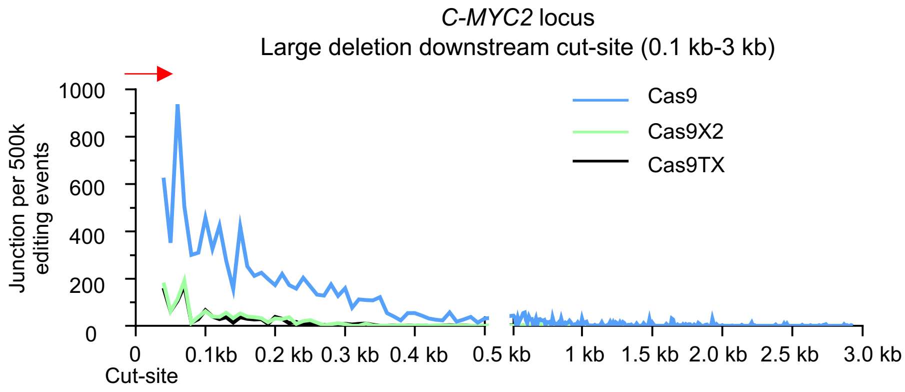

Supplementary Figure 4. Cas9TX reduces chromosomal translocations and large deletions
a. SDS PAGE showing purified TREX2, TREX2-3R, Cas9X2, and Cas9TX. **b**. Digestion of a 38-mer oligo by TREX2 and TREX2-3R via the *in vitro* cleavage assay. **c** and **d**. Junction bias of *Sp*Cas9, Cas9X2, and Cas9TX at the off-target sites of *HBA1* (**c**) or *C-MYC2* (**d**) in HEK293T cells detected by PEM-seq. Red numbers highlight sites with too few junctions. **e**. Linear plots of chromosomal deletion junctions within the 0.1 to 3 kb region downstream of the *C-MYC2* locus per 500,000 total editing events detected by PEM-seq in HEK293T cells. The red arrow indicates the position of the PEM-seq primer. Source data are provided as a Source Data file.

a

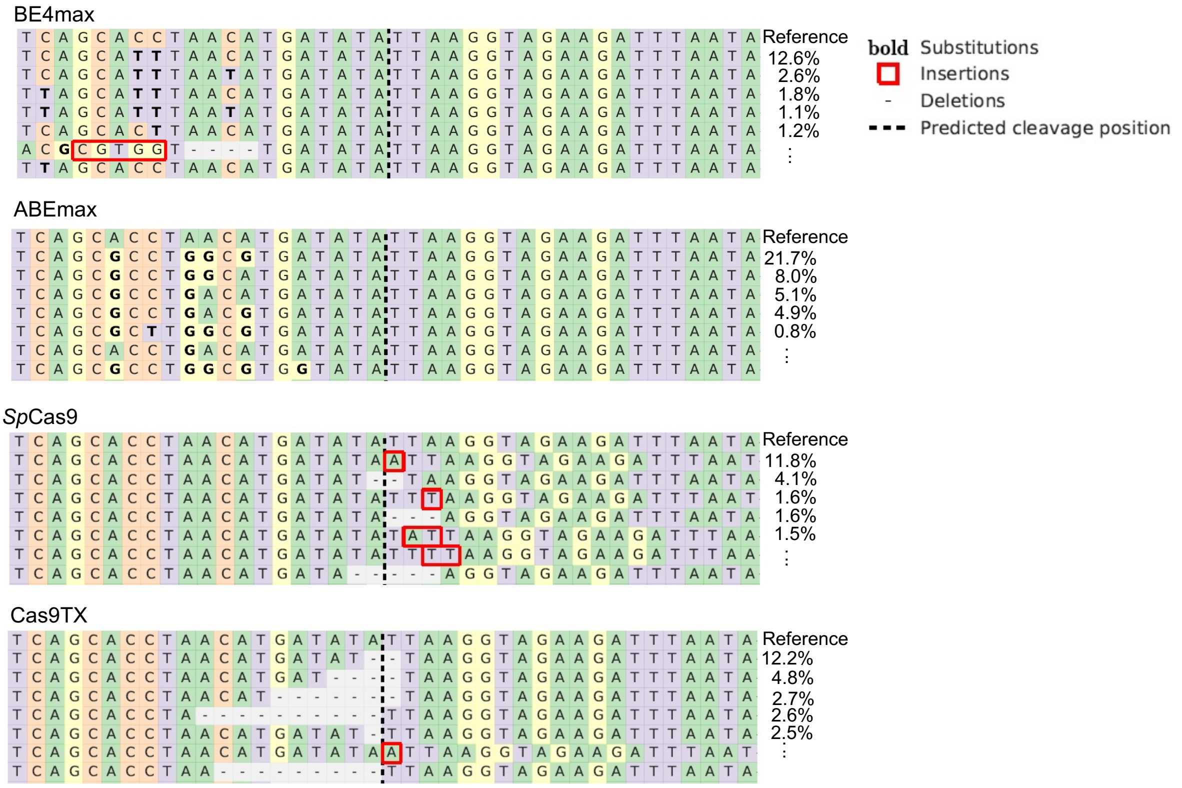

b

General translocation junctions for *RAG1C*

	*Sp*Cas9	Cas9TX	BE4max	ABEmax	Control
Translocations	913	279	125	122	15
Editing events	46k	65k	57k	84k	0.2k
Total events	91k	126k	289k	212k	249k

c

General translocations

	*Sp*Cas9	Cas9TX	BE4max	ABEmax
EMX1	1.21%	0.14%	0.16%	-
DNMT1-2	1.24%	0.24%	0.04%	0.02%
C-MYC2	2.98%	0.22%	0.11%	-
RAG1C	1.93%	0.42%	0.22%	0.15%
BCL11A	1.75%	0.43%	0.11%	0.07%

d

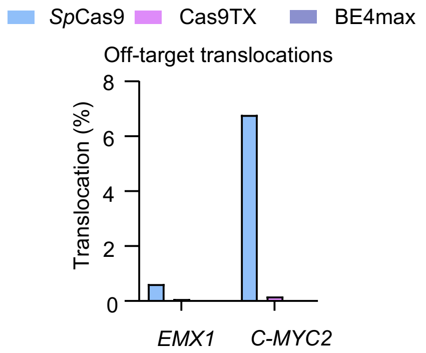

e

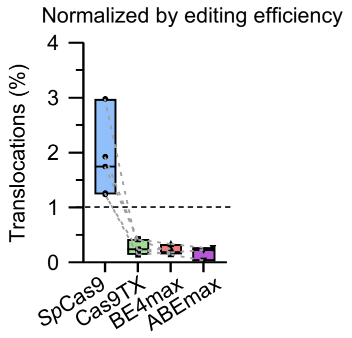

Supplementary Figure 5. Cas9TX reduces chromosomal translocations to the level of base editors

a. Editing outcomes of BE4max, ABEmax, *Sp*Cas9, and Cas9TX at the *RAG1C* locus detected by PEM-seq. Percentages are shown on the right. **b**. Junction numbers of general translocations, editing events, and total sequencing events for BE4max, ABEmax, *Sp*Cas9, Cas9TX, and the un-cut control at the *RAG1C* locus detected by PEM-seq. **c**. Percentages of general translocations for *Sp*Cas9, Cas9TX, BE4max, and ABEmax at *EMX1, C-MYC2*, *DNMT1-2*, *RAG1C,* and *BCL11A* in HEK293T cells detected by PEM-seq. "-" indicates that related base editors are not applied in this locus for the sequence context. **d**. Percentages of off-target translocations for *Sp*Cas9, Cas9TX, and BE4max at *EMX1* and *MYC2* in HEK293T cells detected by PEM-seq. **e**. Percentages of general translocations of *Sp*Cas9, Cas9TX, BE4max, and ABEmax normalized to editing efficiency at *EMX1*, *C-MYC2*, *DNMT1-2*, *RAG1C,* and *BCL11A* in HEK293T cells, one independent PEM-seq library for each locus, N=5. Values from minimum to maximum are shown in the box. The line represents the median.

a

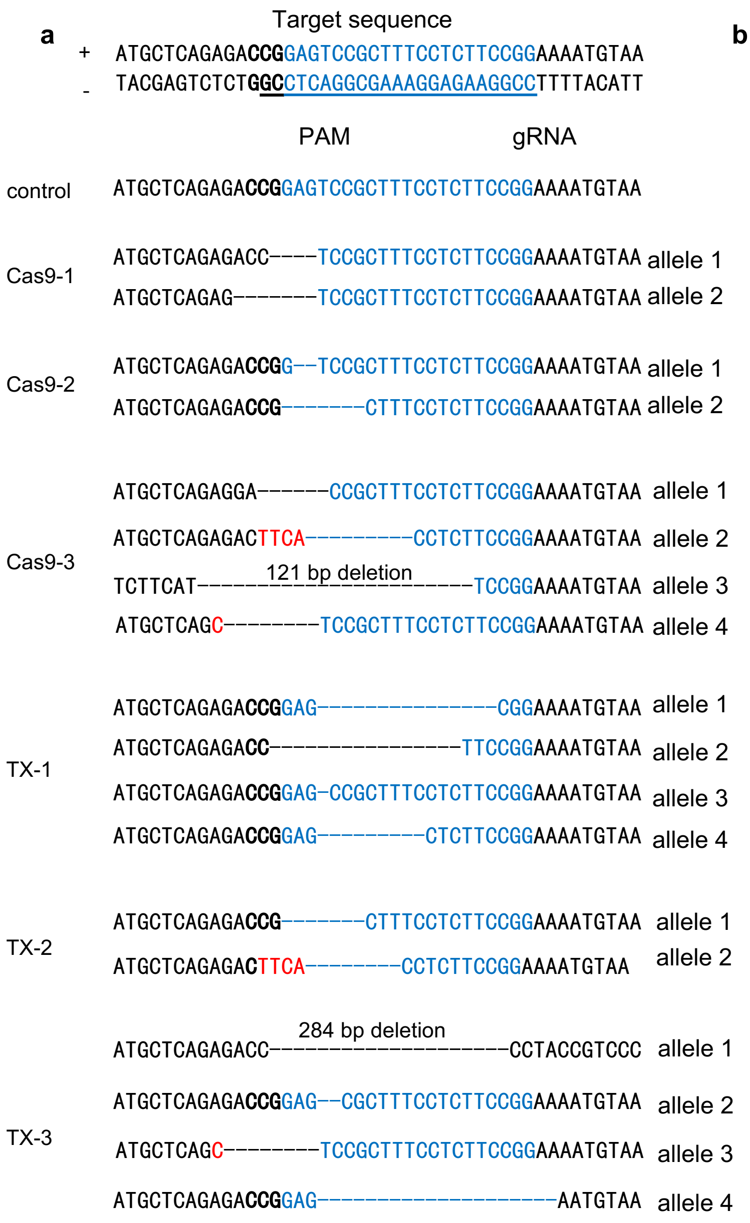

b

Libraries	Reads
Control-1	64,485,614
Control-2	75,423,332
Control-3	76,727,474
Cas9-1	69,768,959
Cas9-2	71,547,392
Cas9-3	41,284,196
Cas9TX-1	82,824,949
Cas9TX-2	107,204,400
Cas9TX-3	47,314,594

Supplementary Figure 6. WGS analysis for Cas9 and Cas9TX-edited mESCs

a. Sequences for *Wrp53* locus edited by Cas9 and Cas9TX identified by Sanger sequencing. Note that Cas3-3 and Cas9TX-1 and Cas9TX-3 have 4 edited alleles. This may be because transfection was done in metaphase or more than one clones were sorted into one well. **b**. Total WGS reads for each clone.

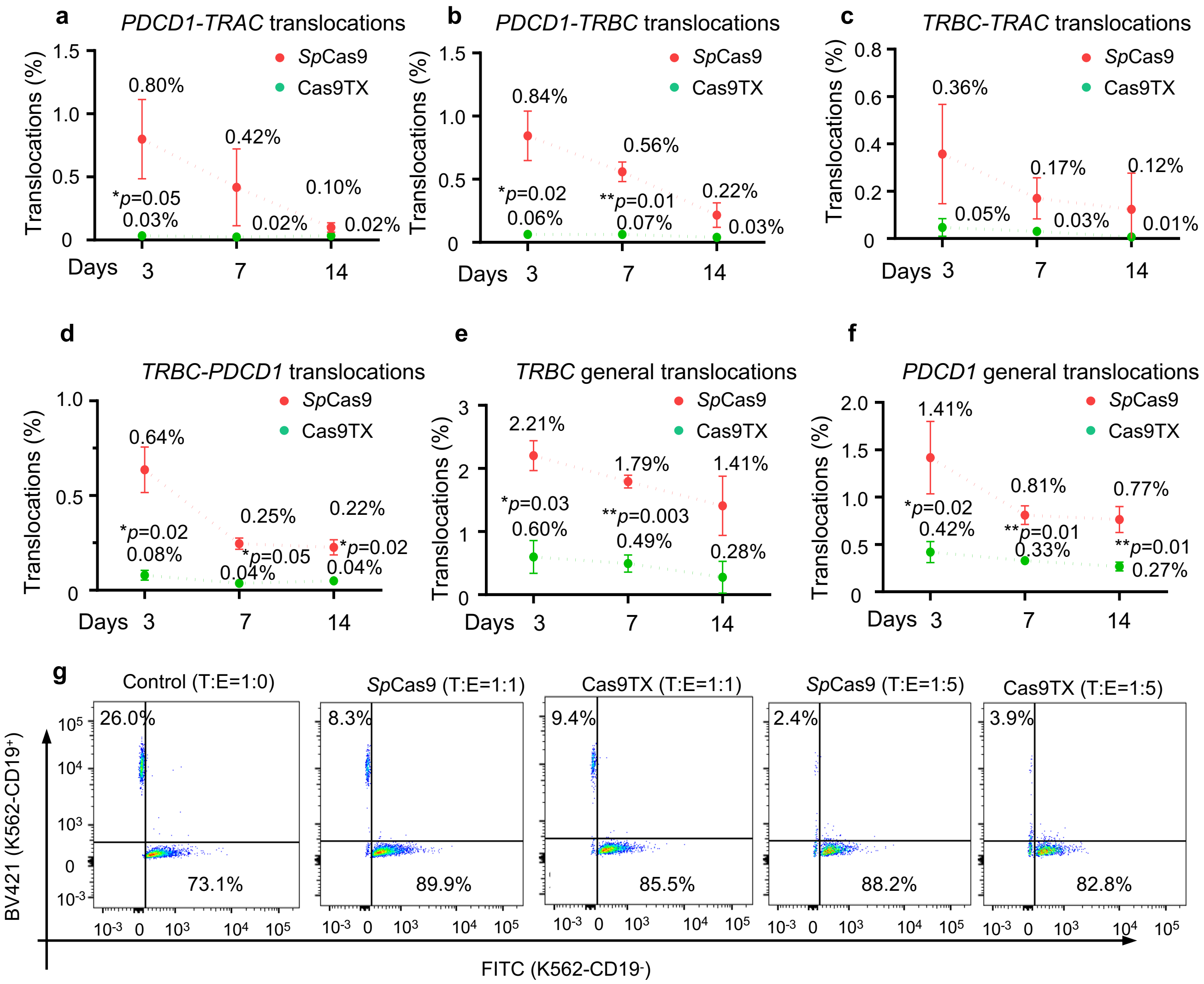

Supplementary Figure 7. Cas9TX nearly eliminates chromosomal translocations in CAR T cells

a-f. Translocation ratios of *PDCD1-TRAC* (**a**), *PDCD1-TRBC* (**b**), *TRBC-TRAC* (**c**), *TRBC-PDCD1* (**d**), *TRBC* general translocations (**e**), and *PDCD1* general translocations (**f**) for *Sp*Cas9 and Cas9TX in CAR T cells at 3 days, 7 days and 14 days post-transfection detected by PEM-seq. Mean ± SD from three replicates, and the mean value is shown on the top of each point. Two-tailed *t*-test, $*p < 0.05$, $**p < 0.01$. (**g**). Percentages of $CD19^+$ and $CD19^-$ K562 control cells co-cultured with CAR-T cells at the indicated ratios detected by FACS at 24 hours post mixing. $CD19^+$ K562 cells carry BFP and $CD19^-$ K562 cells carry GFP.

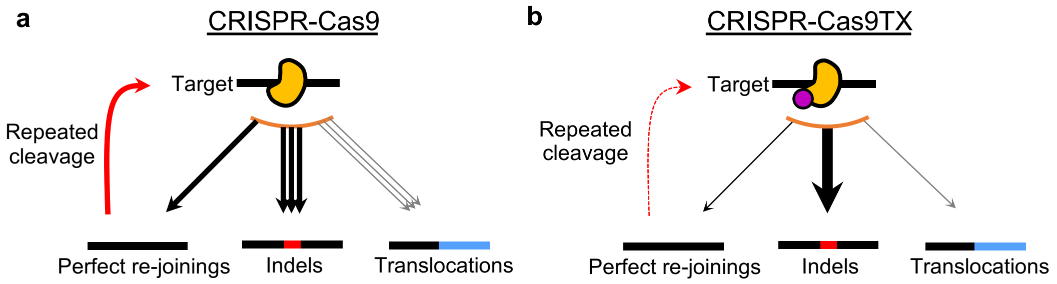

Supplementary Figure 8. Models for the elimination of chromosomal translocations by Cas9TX
a. Perfect re-joinings, indels, and translocations are generated after each round of *Sp*Cas9 (yellow) cleavage. The perfect repair products can be repeatedly cleaved by *Sp*Cas9, and indels and translocations accumulate after each round of cleavage. **b**. Cas9TX inhibits chromosomal translocations by enhancing the generation of indels that are mainly resistant to repeated cleavage (purple) to reduce the number of perfect re-joinings.

Supplementary Table 1. PEM-seq data in T cells

PEM-seq in Figure1									
	3days			7 days			14 days		
Locus: TRAC	CC071a	CC071b	CC072a	CC074a	CC074b	CC075a	CC078a	CC077a	CC077b
	Cas9	Cas9	Cas9	Cas9	Cas9	Cas9	Cas9	Cas9	Cas9
Translocation	1,064	1,567	758	1,409	956	748	272	938	557
Editing Events	83,055	116,187	75,205	151,240	98,552	82,832	44,488	118,318	59,665
Total Events	141,276	222,496	235,219	303,193	227,984	326,926	337,467	448,930	216,664
Editing Efficiency	58.8%	52.2%	32.0%	49.9%	43.2%	25.3%	13.2%	26.4%	27.5%
TRBC	256	329	85	252	212	75	13	165	73
PDCD1	411	479	99	330	228	68	20	190	129
TRAC-OT	8								
Locus: PDCD1	CC080a	CC080b	CC080c	CC081a	CC081b	CC081c	CC082a	CC082b	CC082c
Translocation	3,298	3,575	1,873	3,288	2,590	1,362	1,880	1,398	503
Editing Events	83,282	102,244	60,481	113,806	106,047	77,688	81,257	80,479	37,488
Total Events	107,796	148,294	130,011	181,660	177,728	216,612	184,041	199,050	137,404
Editing Efficiency	77.3%	68.9%	46.5%	62.6%	59.7%	35.9%	44.2%	40.4%	27.3%
TRAC	1,165	989	344	749	663	169	452	248	21
TRBC	1,334	1,425	511	1,287	1,024	334	697	534	92
TRAC-OT		4							
Locus: TRBC	CC090a	CC090b	CC090c	CC092a	CC092b	CC092c	CC094a	CC094b	CC094c
Translocation	49	617	361	469	191	216	298	220	162
Editing Events	1,290	18,040	12,565	18,110	9,262	10,702	16,416	12,098	7,379
Total Events	2,065	31,364	32,349	38,509	21,107	37,904	60,122	44,723	43,598
Editing Efficiency	62.5%	57.5%	38.8%	47.0%	43.9%	28.2%	27.3%	27.1%	16.9%
TRAC-OT	1	0	1	1	0	1	3	0	0
TRAC	11	106	23	68	20	9	21	16	3
PDCD1	8	183	61	115	45	26	63	54	19
Locus: TRAC	YJ339b	YJ339c	YJ340c	YJ339e	TRAC-OT	YJ395b	YJ395c	YJ395d	
	eSp	Fe	HF1	Hypa		Cas9	Cas9	Cas9	
Translocation	3,714	3,776	3,299	3,784		102	145	85	
Editing Events	205,566	263,197	180,642	206,361		2699	4341	1841	
Total Events	498,859	574,635	584,270	430,941		241913	224062	172463	
Editing Efficiency	41.2%	45.8%	30.9%	47.9%		1.1%	1.9%	1.1%	
TRBC	366	351	378	346					
PDCD1	391	362	279	303					
TRAC-OT	0	0	0	0					

Supplementary Table 2. PEM-seq data for junction bias detection for *RAG1A* locus in HEK293T cells

Cell lines	HEK293T-Figure 2			HEK293T- Supplementary Figure 2		
Replicate	1	2	3	1	2	3
Locus	RAG1A			RAG1A		
OT1 bias	2.7	2.8	2.7	0.4	0.2	0.1
Re-targetable	601	407	482	28	41	10
Un-targetable	1,623	1,128	1,323	70	267	99
OT2 bias	3.6	3.0	2.8	0.6	0.4	0.6
Re-targetable	257	275	329	27	10	17
Un-targetable	927	829	931	44	27	27
OT3 bias	2.2	2.2	2.6	0.4	0.1	0.2
Re-targetable	241	151	157	39	51	26
Un-targetable	535	336	403	91	364	135
OT4 bias	3.1	3.0	4.1	0.5	0.2	0.3
Re-targetable	220	159	153	32	23	31
Un-targetable	671	482	622	63	133	113
OT5 bias	3.9	4.3	3.8	0.4	0.2	0.2
Re-targetable	63	51	77	85	173	57
Un-targetable	248	219	296	202	789	293
OT6 bias	3.2	4.5	5.6	0.2	0.2	0.2
Re-targetable	121	52	74	36	63	48
Un-targetable	386	234	416	169	314	256
OT1 sequence	TCCTCCTCCCCACCCACCTT CAG					
OT2 sequence	ACGTCTTCCCCACCCACCTG GGG					
OT3 sequence	CCCTCCTTCCCACCCACTTT GGG					
OT4 sequence	ACTTCCTTCCCACCCACCTT CAG					
OT5 sequence	TCCTCTTCTCCACCCACCTC TGG					
OT6 sequence	GCCTCTTCCACACCCACCCT GGG					

Supplementary Table 3. PEM-seq data for junction bias detection for other loci in HEK293T cells

Cell lines	HEK293T-Figure 2								
Replicate	1	2	3	1	2	3	1	2	3
Locus	RAG1B			DNMT1			MYC1		
OT1 bias	1.9	1.8	2.2	0.6	1.6	1.0	2.0	1.7	2.3
Re-targetable	244	224	109	10	14	30	46	56	90
Un-targetable	473	414	238	6	23	31	94	97	211
OT2 bias	2.8	0.8	1.4	4.0	2.0	1.7	1.8	1.1	1.5
Re-targetable	26	39	18	4	29	17	43	69	78
Un-targetable	73	30	25	16	57	29	77	79	114
OT3 bias							3.1	4.5	3.1
Re-targetable							14	10	23
Un-targetable							44	45	71
OT4 bias							2.5	3.4	2.5
Re-targetable							28	19	55
Un-targetable							71	64	138
OT1 sequence	TCCTTGTTTTCATTGTTCT C TGG			TTCATGGCAGATGTTTA CCT GGG			GGGGTGTGGAGCTTGAC TAT GAG		
OT2 sequence	CATTTGTTTTCATTGTTCT C TGG			GTCCAGGCAGAAGTTTA CCT GGG			AGGATGAAGAGATTGGC TAT GGG		
OT3 sequence							GGGAAGTGGAACCTGGC TCT GGG		
OT4 sequence							TGGATGTGCAGCCTGGC TAT TGG		

Supplementary Table 4. PEM-seq data for Cas9 fused with exonucleases

	YJ380a	YJ380b	YJ380c	YJ380d	YJ380e	YJ380f	YJ380g	YJ380h
	Cas9	TREX2	TREX1	T5	Artemis	CTIP	Mre11	Exo1
Editing Events	271,581	270,036	384,373	190,894	319,031	225,703	397,000	244,409
Total Events	443,387	348,710	503,326	273,280	509,052	383,141	582,093	461,625
Editing Efficiency	47.8%	75.9%	74.9%	63.8%	46.1%	44.3%	50.8%	42.6%
OT-translocation	16,471	413	337	3,577	24,717	12,073	27,880	12,632
OT-	1,996	169	104	898	2824	1,328	3,832	1,600
OT+	14,474	244	233	2,679	21,893	10,745	24,047	11,032
bias	7.3	1.4	2.2	3.0	7.8	8.1	6.3	6.9

Supplementary Table 5. PEM-seq data for Cas9, Cas9X2, T2A-TREX2, and Cas9X2d

	DNMT2				EMX1				DNMT1			
	Cas9	t2a-x2	Cas9X2	Cas9X2d	Cas9	t2a-x2	Cas9X2	Cas9X2d	Cas9	t2a-x2	Cas9X2	Cas9X2d
	YJ356a	YJ356b	YJ356c	YJ356d	YJ357a	YJ357b	YJ357c	YJ357d	CC104a	CC104b	CC104c	CC104d
Editing Events	68,369	27,881	62,647	39,384	54,361	101,926	70,552	79,803	110,740	120,783	119,142	58,657
Total Events	113,014	39,957	83,924	104,348	71,480	130,009	109,216	150,295	177,637	209,204	234,859	232,085
General translocation	2,245	649	643	1,798	327	361	149	743	2045	1,099	553	1,505
OT translocation	0	0	0	0	118	44	0	229	16	6	5	2
	RAG1A				RAG1B				RAG1C			
	Cas9	t2a-x2	Cas9X2	Cas9X2d	Cas9	t2a-x2	Cas9X2	Cas9X2d	Cas9	t2a-x2	Cas9X2	Cas9X2d
	YJ362a	YJ362b	YJ362c	YJ362d	YJ363a	YJ363b	YJ363c	YJ363d	YJ364a	YJ364b	YJ364c	YJ364d
Editing Events	93,366	35,813	73,881	21,645	34,016	42,959	21,880	4,806	59,554	45,858	38,796	5,984
Total Events	253,395	97,208	268,876	164,967	148,916	158,666	109,574	52,033	228,699	145,410	229,621	87,810
General translocation	3,145	599	603	1,126	2,023	889	287	626	1,621	621	264	197
OT translocation	681	45	32	96	695	170	10	64	0	0	0	0
	MYC1				MYC2				MYC3			
	Cas9	t2a-x2	Cas9X2	Cas9X2d	Cas9	t2a-x2	Cas9X2	Cas9X2d	Cas9	t2a-x2	Cas9X2	Cas9X2d
	YJ359a	YJ359b	YJ359c	YJ359d	YJ360a	YJ360b	YJ360c	YJ360d	YJ361a	YJ361b	YJ361c	YJ361d
Editing Events	69,693	100,632	125,094	95,793	78,691	126,566	114,284	64,628	134,963	171,043	93,081	53,945
Total Events	84,553	129,011	139,228	162,693	144,239	195,141	164,584	159,126	354,951	366,867	293,345	271,088
General translocation	1,985	1,152	652	1,845	2,865	1,729	580	2,683	2,715	1,232	620	1,826
OT translocation	19	8	3	8	4,203	1,993	160	2,747	30	6	3	6

Supplementary Table 6. PEM-seq data for Cas9 and Cas9TX

	DNMT1		MYC1		DNMT2		MYC3		Pten		P53	
	Cas9	Cas9TX	Cas9	Cas9TX	Cas9	Cas9TX	Cas9	Cas9TX	Cas9	Cas9TX	Cas9	Cas9TX
	YJ365a	YJ365b	YJ366a	YJ366b	YJ367a	YJ367b	YJ370a	YJ370b	YJ371a	YJ371b	YJ380a	YJ380b
Total Events	416,639	271,564	341,139	546,191	226,525	333,771	281,565	349,815	297,498	443,416	371,752	152,855
Editing Efficiency	73.1%	81.5%	70.4%	83.7%	68.6%	82.8%	53.9%	61.3%	75.7%	83.0%	88.1%	88.8%
Tranloslo cation	3,151	677	5,529	1,145	2,203	803	2,854	731	6,508	2,701	1,642	614
General transloc	2,339	615	2,974	983	2,001	695	2,374	649	6,030	2,378	1,425	550
OT translocat ion	62	3	2200	79	7	2	346	9	0	0	0	0
	RAG1B		VEGFA		RAG1C		TRAC		TRBC		PDCD1	
	Cas9	Cas9TX	Cas9	Cas9TX	Cas9	Cas9TX	Cas9	Cas9TX	Cas9TX	Cas9	Cas9TX	Cas9
	YJ381a	YJ381b	YJ372a	YJ372b	YJ373a	YJ373b	YJ376a	YJ376b	YJ377a	YJ377b	YJ378a	YJ378b
Total Events	146,436	127,712	47,949	45,012	82,427	113,894	96,929	119,372	139,539	100,100	105,058	96,583
Editing Efficiency	34.4%	45.3%	26.5%	79.1%	46.7%	49.5%	63.4%	70.5%	71.0%	49.0%	76.7%	64.0%
Tranloslo cation	4,504	707	6,065	683	855	271	2,195	1,055	1,874	5,017	1,589	3,263
General transloc	1,702	466	1,394	281	816	253	1,871	984	1,512	3,127	1,246	2,150
OT translocat ion	2,625	195	4,591	376	0	0	13	1	256	1,359	49	151

Supplementary Table 7. PEM-seq data in CAR T cells

PEM-seq in Figure 7												
	3 days						7 days					
TRAC	YJ274a	YJ274b	YJ275a	YJ275b	CC073a	CC073b	YJ280a	YJ280b	YJ281a	YJ281b	CC076a	CC076b
	Cas9	Cas9TX	Cas9	Cas9TX	Cas9	TX	Cas9	Cas9TX	Cas9	Cas9TX	Cas9	Cas9TX
Translocation	2,991	230	2,090	374	731	167	1,388	122	783	124	561	321
Editing Events	268,078	168,402	193,560	245,524	80,525	63,003	163,370	78,956	131,317	115,370	81,809	166,247
Total Events	411,774	485,612	349,832	631,391	267,319	113,675	407,870	261,528	400,214	365,966	371,759	329,821
Editing Efficiency	65.1%	34.7%	55.3%	38.9%	30.1%	55.4%	40.1%	30.2%	32.8%	31.5%	22.0%	50.4%
TRBC	581	21	360	44	85	20	199	0	93	9	38	15
PDCD1	794	21	477	30	119	9	200	2	78	3	44	25
TRAC-OT	4	0	1	0								
PDCD1	CC083c	CC084c	CC084b	CC084a	CC083a	CC083b	CC086c	CC087a	CC087b	CC087c	CC086a	CC086b
	Cas9	Cas9TX	Cas9	Cas9TX	Cas9	Cas9TX	Cas9	Cas9TX	Cas9	Cas9TX	Cas9	Cas9TX
Translocation	360	31	2,431	421	1,296	140	1,775	352	1,301	235	870	515
Editing Events	9,740	6,670	76,347	71,139	50,999	19,174	81,487	72,158	69,005	46,663	53,249	104,901
Total Events	13,088	14,072	131,769	150,274	112,064	26,058	195,090	242,281	200,233	166,118	148,058	180,002
Editing Efficiency	74.4%	47.4%	57.9%	47.3%	45.5%	73.6%	41.8%	29.8%	34.5%	28.1%	36.0%	58.3%
TRAC-PDCD1	92	1	776	33	222	8	533	18	367	14	36	17
TRBC	84	2	783	48	326	18	532	35	343	30	281	88
TRAC-OT												
TRBC	CC091c	CC096c	CC096b	CC096a	CC091a	CC091b	CC093c	CC097a	CC097b	CC097c	CC093a	CC093b
	Cas9	Cas9TX	Cas9	Cas9TX	Cas9	Cas9TX	Cas9	Cas9TX	Cas9	Cas9TX	Cas9	Cas9TX
Translocation	522	69	434	74	404	97	191	41	160	23	207	180
Editing Events	15,981	10,245	12,769	11,474	12,889	9,178	7,796	6,304	6,879	4,223	9,379	23,248
Total Events	29,907	28,795	29,812	32,467	33,875	14,543	31,411	31,127	33,032	17,591	35,948	45,538
Editing Efficiency	53.4%	35.6%	42.8%	35.3%	38.0%	63.1%	24.8%	20.3%	20.8%	24.0%	26.1%	51.1%
TRAC	83	3	55	10	16	2	16	1	16	1	7	11
PDCD1	117	5	87	10	65	9	19	1	19	2	21	10
TRAC-OT	1	0	3	0	0	0	0	0	0	0	0	0
	14 days-TRAC						14 days-PDCD1					
	YJ304a	YJ304b	YJ305a	YJ305b	CC079a	CC079b	CC088c	CC089a	CC089b	CC089c	CC088a	CC088b
	Cas9	Cas9TX	Cas9	Cas9TX	Cas9	Cas9TX	Cas9	Cas9TX	Cas9	Cas9TX	Cas9	Cas9TX
Translocation	449	55	249	96	224	156	111	124	263	124	824	327
Editing Events	76,499	53,704	44,576	55,585	36,607	72,383	8,026	25,863	27,290	24,718	59,002	67,778
Total Events	829,923	606,138	521,986	698,799	290,508	265,057	45,555	177,681	172,147	200,168	220,059	160,145
Editing Efficiency	9.2%	8.9%	8.5%	8.0%	12.6%	27.3%	17.6%	14.6%	15.9%	12.3%	26.8%	42.3%
TRAC	21	2	12	0	15	5	7	8	20	12	83	16
PDCD1	62	1	24	0	11	2	19	6	31	9	179	41
	14 days-TRBC											
TRBC	CC095c	CC098a	CC098b	CC098c	CC095a	CC095b						
	Cas9	Cas9TX	Cas9	Cas9TX	Cas9	Cas9TX						
Translocation	52	13	46	25	148	116						
Editing Events	3,263	2,218	1,690	3,163	7,574	18,387						
Total Events	49,957	28,177	26,015	45,054	46,496	53,830						
Editing Efficiency	6.5%	7.9%	6.5%	7.0%	16.3%	34.2%						
TRAC	1	0	5	0	3	4						
PDCD1	8	1	3	2	19	8						

Supplementary Table 8. Sequence for primers in this study

	Biotin-primer	Nested primer
RAG1A/B	AGGACTGCTGGAGATTGCTC	TATGTGGGTGCTGAATTTCATC
RAG1C	CCTGAGAACAATGAAAACAAGTC	TATCAATATCCCACTGATGTATC
DNMT1-1	GCCCGCACTGAATGCACTTGGGAGG GTG	GCAGGAACACAGATGATGGC
DNMT1-2	GAGAGGCCTCGTTAGGAGCTCTCCT TTG	CTGAATTAAACAGTACCATGTTC
C-MYC1	GGTGACTCACTTGGGAATCGGGAAG GTG	CCTCAGAATAGGAGAGAGTG
C-MYC2	GCCTTGTATTTGTACAGCATTAATCT GG	GGAGGAACAAGAAGATGAGGAAG
C-MYC3	TTGCGACTCTCAGCTGAATCCACTG CTG	TGCTCCCATGGCATCATGAC
EMX1	CCCATCAGGCTCTCAGCTCAGCCTG AGTGTTGAG	CCCAGGTGAAGGTGTGGTTC
HBA1	TCTGGGTCGAGGGGCGAGATG	GGGTTGCGGGAGGTGTAGCG
PTEN	ACGCCCTTGGAGGTTAATCCTC	TCCCAGGTCCGAGACGGTCC
TP53	CAAGCCCAGCGACAGCCGCTG	TCCTTGACTCTGGCAACTGGG
TRAC	CCTGCCGTGTACCAGCTGAGAGACT C	GTGTCACAAAGTAAGGATTCTG
TRBC	CTAGTCTTGTCTGCTACCTGGATC	AGGCCCCACTCACCTGCTCT
PDCD1	GCACCCTCCCTTCAACCTGACCTGG GAC	GAGAAGGCGGCACTCTGGTG
BCL11A	CACACGGCATGGCATACAAATTATTT C	CTGGGCAAACGGCCACCGATG
HBG	CCTCTGTGAAATGACCCATGGCGTC	GGTGCTTCCTTTTATTCTTC
TRAC-F:GTGTACCAGCTGAGAGACTC		
OT-DN-R:GGCATAGCCAATCCATTCAGTGATC		
OT-UP-F:TTTAGCATTTACTCAAAAGTCCACAATCCA		
TRAC-R: TGGTGGCAATGGATAAGGCC		
OT-DN-F: AACTGTGCTAGACATGAGGTACTTCATGTCTTGCATCTGGGTCA		
OT-UP-R: AACTGTGCTAGACATGAGGTCTAAGGAGATCATTTTGGAGTTTTAAGATC		

Supplementary Table 9. gRNA sequence in this study

Locus	sequence
RAG1A	GCCTCTTTCCCACCCACCTT GGG
RAG1B	GACTTGTTTTCATTGTTCTC AGG
RAG1C	GCACCTAACATGATATATTA AGG
DNMT1	TTCCCGGCAGATGTTTACCT TGG
DNMT2	CCCTGCAGTTCCCTAACTGA GGG
C-MYC1	GCTTGGCTATGGGAATAGAA AGG
C-MYC2	GAGTCTGGATCACCTTCTGC TGG
C-MYC3	GTACATGCAGTTCTGCATCT TGG
EMX1	GAGTCCGAGCAGAAGAAGAA GGG
HBA1	GTGCAGAGAAGAGGGTCAGT GGG
PTEN	GGTGAGTAGCTGGTTCCCGT GGG
TP53	GACCATTACTCAGCTCTGAG GGG
TRAC	TGTGCTAGACATGAGGTCTA TGG
TRBC	GGAGAATGACGAGTGGACCC AGG
PDCD1	GGCGCCCTGGCCAGTCGTCT GGG
BCL11A	TTTATCACAGGCTCCAGGAA GGG
HBG	GGTGCTTCCTTTTATTCTTC AGG
