# Supplementary material for: Cas9 exo-endonuclease eliminates chromosomal translocations during genome editing
Source: Nat Commun. 2022 Mar 8;13:1204. doi: 10.1038/s41467-022-28900-w (PMC8904484; doi:10.1038/s41467-022-28900-w)
Supplement: Supplementary file 2 — Reporting Summary [file 41467_2022_28900_MOESM2_ESM.pdf]

nature portfolio

Corresponding author(s): Jiazhi Hu
Last updated by author(s): Feb 10, 2022

Reporting Summary

Nature Portfolio wishes to improve the reproducibility of the work that we publish. This form provides structure for consistency and transparency in reporting. For further information on Nature Portfolio policies, see our Editorial Policies and the Editorial Policy Checklist.

Statistics

For all statistical analyses, confirm that the following items are present in the figure legend, table legend, main text, or Methods section.

n/a	Confirmed	
☐	☒	The exact sample size (*n*) for each experimental group/condition, given as a discrete number and unit of measurement
☐	☒	A statement on whether measurements were taken from distinct samples or whether the same sample was measured repeatedly
☐	☒	The statistical test(s) used AND whether they are one- or two-sided<br>*Only common tests should be described solely by name; describe more complex techniques in the Methods section.*
☐	☒	A description of all covariates tested
☐	☒	A description of any assumptions or corrections, such as tests of normality and adjustment for multiple comparisons
☐	☒	A full description of the statistical parameters including central tendency (e.g. means) or other basic estimates (e.g. regression coefficient) AND variation (e.g. standard deviation) or associated estimates of uncertainty (e.g. confidence intervals)
☐	☒	For null hypothesis testing, the test statistic (e.g. *F*, *t*, *r*) with confidence intervals, effect sizes, degrees of freedom and *P* value noted<br>*Give P values as exact values whenever suitable.*
☒	☐	For Bayesian analysis, information on the choice of priors and Markov chain Monte Carlo settings
☒	☐	For hierarchical and complex designs, identification of the appropriate level for tests and full reporting of outcomes
☒	☐	Estimates of effect sizes (e.g. Cohen's *d*, Pearson's *r*), indicating how they were calculated

Our web collection on statistics for biologists contains articles on many of the points above.

Software and code

Policy information about availability of computer code

Data collection	Next-generation data was collected and demutiplexed by Illumina Hiseq X-Ten and Genewiz platform.<br>FACS data was obtained from BD LSRFortessa.
Data analysis	All custom code used for PEM-seq analysis are available: https://github.com/liumz93/PEM-Q<br>Indel pattern for comparison for Cas9, Cas9TX and base editors was analyzed by CRISPResso (version 1.0.8).<br>WGS data was analyzed by Strelk2 pipeline (version 2.8.2).<br>DNA electrophoresis was showed by Image J (version 1.51 J8).<br>FACS data was analyzed only by FlowJo (version X 10.0.7).

For manuscripts utilizing custom algorithms or software that are central to the research but not yet described in published literature, software must be made available to editors and reviewers. We strongly encourage code deposition in a community repository (e.g. GitHub). See the Nature Portfolio guidelines for submitting code & software for further information.

Data

Policy information about availability of data

All manuscripts must include a data availability statement. This statement should provide the following information, where applicable:
- Accession codes, unique identifiers, or web links for publicly available datasets
- A description of any restrictions on data availability
- For clinical datasets or third party data, please ensure that the statement adheres to our policy

Original and processed PEM-seq data were deposited into the NCBI Gene Expression Omnibus (GEO) (GSE116231) and the National Omics Data Encyclopedia

(NODE) (OEP000911). Other original and processed data are in the figures and supplemental materials of this manuscript. Code for PEM-seq analysis is available at the GitHub site: https://github.com/liumz93/PEM-Q.

Field-specific reporting

Please select the one below that is the best fit for your research. If you are not sure, read the appropriate sections before making your selection.

[x] Life sciences   [ ] Behavioural & social sciences   [ ] Ecological, evolutionary & environmental sciences

For a reference copy of the document with all sections, see nature.com/documents/nr-reporting-summary-flat.pdf

Life sciences study design

All studies must disclose on these points even when the disclosure is negative.

Sample size	No sample size calculation was performed. Based on previous studies, at least three replicates or one PEM-seq analysis at at least 3 different loci were performed for experiments in T cells and cell lines.
Data exclusions	No data was excluded.
Replication	All replication attempts were successful and many different gRNAs were tested in different cell lines to ensure the robustness. Moreover, our findings on indel pattern by PEM-seq analysis for Cas9X2 was consistent with previous results published by other groups.
Randomization	Randomization was not performed due to the small sample size of replicates.
Blinding	No blinding was performed due to the obvious effect of Cas9TX. Moreover, PEM-seq analysis can self confirm which nuclease edited the sample.

Reporting for specific materials, systems and methods

We require information from authors about some types of materials, experimental systems and methods used in many studies. Here, indicate whether each material, system or method listed is relevant to your study. If you are not sure if a list item applies to your research, read the appropriate section before selecting a response.

Materials & experimental systems

n/a	Involved in the study	
[ ]	[x]	Antibodies
[ ]	[x]	Eukaryotic cell lines
[x]	[ ]	Palaeontology and archaeology
[x]	[ ]	Animals and other organisms
[x]	[ ]	Human research participants
[x]	[ ]	Clinical data
[x]	[ ]	Dual use research of concern

Methods

n/a	Involved in the study	
[x]	[ ]	ChIP-seq
[ ]	[x]	Flow cytometry
[x]	[ ]	MRI-based neuroimaging

Antibodies

Antibodies used	PE/Cyanine7 mouse anti-human TCR α/β (1:100) (BioLegend, cat.no.306719)<br>Mouse anti-human CD3/28 (Thermo Fisher, cat. no.11161D)(ratio for numbers of beads to cells=1:1)<br>FITC mouse anti-BrdU (1:100)(BD Biosciences, cat.no.556028)<br>Rabbit anti-γH2A.X (1:500)(Abcam, cat.no.ab2893)<br>Alexa 488 Fluor Goat anti-Rabbit IgG HRP(1:500)(Abcam, cat.no. ab6721)
Validation	Validation for Biolegend antibodies can be found at: https://www.biolegend.com;<br>Validation for anti-BrdU can be found at: https://www.bdbiosciences.com/en-us/products/reagents/flow-cytometry-reagents/research-reagents/panels-multicolor-cocktails-ruo/fitc-mouse-anti-brdu-set.556028<br>Validation for anti-γH2A.X can be found at: https://www.abcam.com/gamma-h2ax-phospho-s139-antibody-ab2893.html;<br>Validation for Goat anti-Rabbit IgG HRPcan be found at: https://www.abcam.com/Goat-Rabbit-IgG-HL-Alexa-Fluor-488-ab150077.html

Eukaryotic cell lines

Policy information about cell lines

Cell line source(s)	K562 from National Infrastructure of Cell line resource (China)<br>HEK293T, mESC from ATCC
Authentication	Cell lines were confirmed by STR (short tandem repeat).
Mycoplasma contamination	Mycoplasma contamination was negative.
Commonly misidentified lines<br>(See ICLAC register)	No

Flow Cytometry

Plots

Confirm that:

- [x] The axis labels state the marker and fluorochrome used (e.g. CD4-FITC).
- [x] The axis scales are clearly visible. Include numbers along axes only for bottom left plot of group (a 'group' is an analysis of identical markers).
- [x] All plots are contour plots with outliers or pseudocolor plots.
- [x] A numerical value for number of cells or percentage (with statistics) is provided.

Methodology

Sample preparation	Cells were isolated with or without Trypsin followed by centrifugation. PBS were used for washing and cells were resuspended in PBS with 2% FBS followed by FACS analysis.
Instrument	BD LSRFortessa, MoFlo, Aria SORP
Software	FlowJo X (version 10.0.7)
Cell population abundance	Cell population were gated for top 30%-40% in HEK293T cells. For car T kill assay, cells with relevant fluorescence were gated for the obvious clustering.
Gating strategy	A lymphocyte gate was defined from FSC-A v SSC-A. Additional gating was executed as described in figure and figure legends for individual experiments.

- [x] Tick this box to confirm that a figure exemplifying the gating strategy is provided in the Supplementary Information.
